# Physical activity and renal function in the Italian kidney transplant population

**DOI:** 10.1080/0886022X.2020.1847723

**Published:** 2020-11-30

**Authors:** Lucia Masiero, Francesca Puoti, Lia Bellis, Letizia Lombardini, Valentina Totti, Maria Laura Angelini, Alessandra Spazzoli, Alessandro Nanni Costa, Massimo Cardillo, Gianluigi Sella, Giovanni Mosconi

**Affiliations:** a Italian National Transplant Center, Rome, Italy; b Department of Biomedical & Neuromotor Sciences, University of Bologna, Bologna, Italy; c ANED, Milan, Italy; d Nephrology and Dialysis Unit, Morgagni-Pierantoni Hospital, AUSL Romagna, Forlì, Italy; e Sport Medicine Unit, AUSL Romagna, Ravenna, Italy

**Keywords:** eGFR, exercise, kidney transplant recipients, mixed-regression-analysis, physical activity, propensity score match, renal function, sedentary lifestyle

## Abstract

**Background:**

The well-documented benefits of physical activity (PA) are still poorly characterized in long-term kidney transplant outcome. This study analyzed the impact over a 10-year follow-up of PA on graft function in Italian kidney transplant recipients (KTRs).

**Methods:**

Since 2002, the Italian Transplant-Information-System collected donor and recipient baseline and transplant-related parameters in KTRs. In 2015, ‘penchant for PA’ (PA ≥ 30-min, 5 times/week) was added. Stable patients aged ≥18 years at the time of first-transplantation were eligible. KTRs with at least 10-year follow-up were also analyzed. Mixed-effect regression models were used to compare eGFR changes over time in active versus non-active patients.

**Results:**

PA information was available for 6,055 KTRs (active 51.6%, non-active 48.4%). Lower penchant for PA was found in overweight and obese patients (OR = 0.84; OR = 0.48, respectively), in those with longer dialysis vintage (OR = 0.98 every year of dialysis), and older age at transplant. Male subjects showed greater penchant for PA (OR = 1.25). A slower decline of eGFR over time was observed in active KTRs compared to non-active, and this finding was confirmed in the subgroup with at least 10-year follow-up (n = 2,060). After applying the propensity score matching to reduce confounding factors, mixed-effect regression models corroborated such better long-term trend of graft function preservation in active KTRs.

**Conclusions:**

Penchant for PA is more frequent among male and younger KTRs. Moreover, in our group of Italian KTRs, active patients revealed higher eGFR values and preserved kidney function over time, up to 10-years of follow-up.

## Introduction

Kidney transplantation is the gold standard renal replacement therapy for patients with end stage renal disease (ESRD). Long-term graft function is the result of multiple factors, such as immune-modulation, organ donation features, cardiovascular complications, metabolic disorders and adherence to treatments (regular intake of drugs, lifestyle). Cardiovascular disease (CVD) remains the leading cause of graft loss, morbidity and mortality in kidney transplant recipients (KTRs). Among the main modifiable cardiovascular risk factors, physical inactivity might substantially benefit from appropriate post-transplant interventions [1]. In the general population, sedentary subjects appear to be more susceptible to renal disease (up to an OR = 57%) [2–5], whereas physically active lifestyle in the elderly (≥65 years) has been associated with a reduction by 28% of the risk of renal function deterioration [6,7]. Similarly, low physical activity (PA) and sedentary behavior in KTRs have been proven to negatively affect cardiovascular risk and all-cause mortality [8–14]. On the other hand, regular exercise training commonly results in more favorable clinical outcomes in chronic kidney disease (CKD), as well as after renal transplantation [2,9]. Previous evidence has shown that exercise provides benefits on metabolic profile, overweight and hypertension, factors closely related with clinical, functional and quality of life aspects in transplanted patients [4]. Indeed, PA represents a promising intervention tool for long-term preservation of renal function [3,5,10], although the currently available studies are based on small size populations and short follow-up periods [11,15]. In KTRs, estimated glomerular filtration rate (eGFR) decline over time is an important surrogate marker of death and graft failure [12]. Percentage decrease in eGFR between the first and third year post-transplant is also significantly predictive of patient survival, and a drop in eGFR ≥ 30% has been associated with a 2.2-fold increased mortality in KTRs [13]. Besides the positive effects on graft function preservation [16], PA has been shown to reduce the risk of CVD [17], metabolic disease and diabetes [9,18], and to facilitate a better control of weight gain following successful kidney transplant [19]. To date, there is no evidence of a harmful impact of exercise on renal function, thus, PA can be performed safely during the different stages of CKD and after transplantation [20,21]. Although some studies suggested that intense exercise might increase proteinuria, this is a transient event without enduring adverse rebounds on organ function [22–24].

To the best of our knowledge, the association between PA and renal function has been poorly investigated in the long-term. The aim of this retrospective study was to evaluate the Italian KTR population habits in terms of regular exercise (active and non-active) and to assess whether an association exists between the penchant for PA and the changes in graft function in long-term followed-up KTRs (over 10 years).

## Methods

### The Italian National Transplant Center (CNT) database

The CNT coordinates all the activities related to donation and transplantation of organs, tissues and cells. Through the National Transplant Information System (SIT), the CNT contributes to the management and storage of data, analysis, interpretation of results and dissemination of products. Since 2002, the information related to the entire donation–transplant–follow-up process are compulsorily registered by the Transplant Centers of the National Transplant Network in the SIT [25].

**Figure 1.**
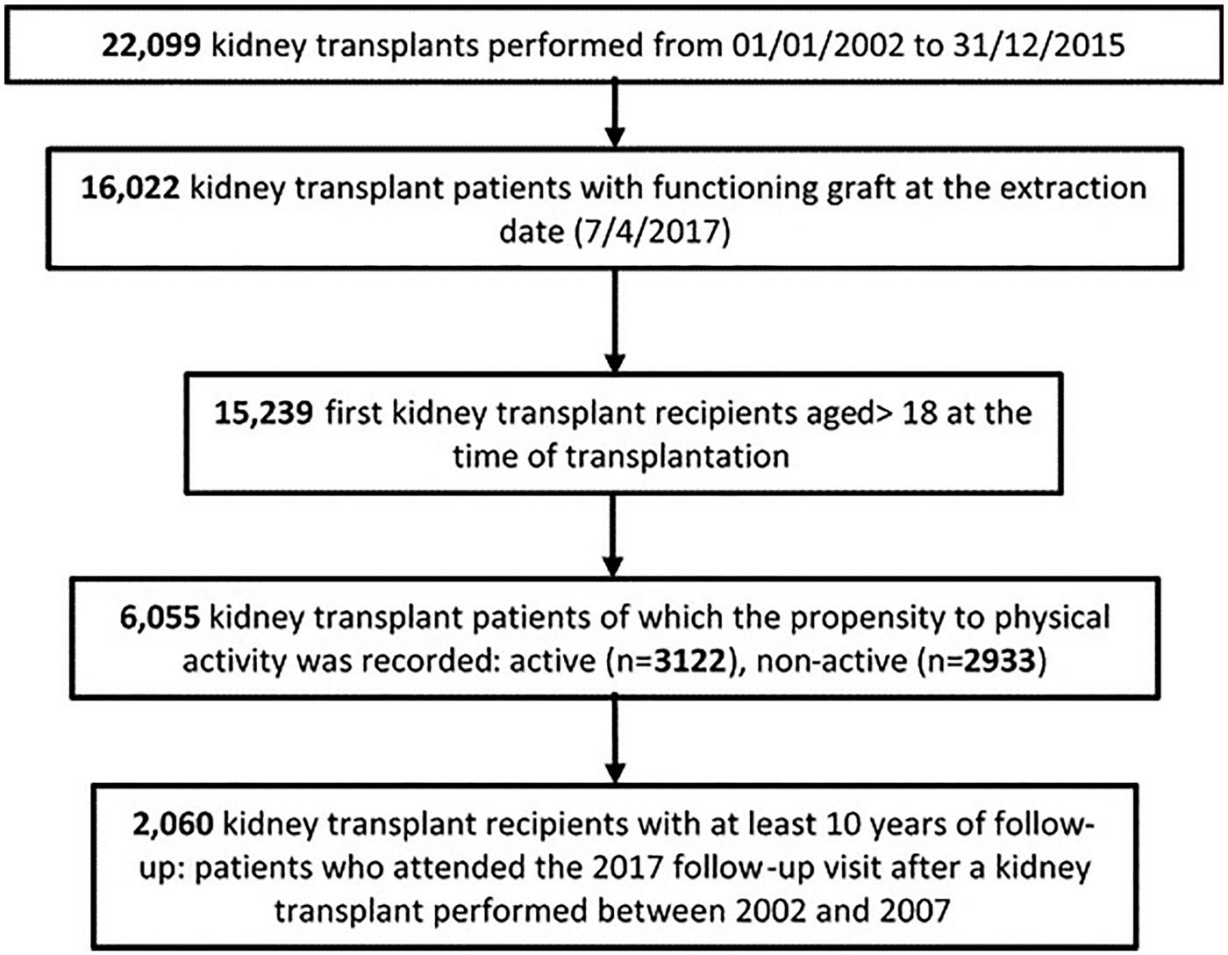
Flow chart of analyzed data selection.

CNT-SIT recorded all 22,099 kidney transplants performed from 01/01/2002 to 31/12/2015. On the data-extraction day (4 April 2017), 16,022 KTRs with a functioning graft (currently defined as no graft failure and no need for dialysis) were identified, with effective annual follow-up (meaning that the patients underwent regularly the follow-up visits until 2017). Among them, 15,239 were eligible for the present analysis: adult subjects (age ≥18 years at transplant) receiving the first kidney transplant (Figure 1).

At the last annual follow-up visit, an additional specific interview was conducted, in order to ascertain the penchant for doing regular PA, defined according to the indications of KDIGO Clinical Practice Guideline 2012 as ‘exercise program compatible with cardiovascular health and tolerance, aiming for at least 30 min 5 times per week’ [26].

For the purpose of this retrospective pilot analysis, we grouped the patients into active (the ones who replied ‘Yes’ to the question about penchant for PA) and non-active (those who replied ‘No’), based on the answer to the question whether he/she was physically active at the follow-up interviews.

Being aware that only alive and functioning allografts can be evaluated and to minimize biases potentially affecting the comparison active versus non-active, we selected a more homogeneous cohort with respect to long-term survival, consisting of a subset of 2,060 KTRs with at least 10-year follow-up (patients transplanted between 2002 and 2007; Figure 1).

### Outcomes and covariates

Primarily, to evaluate the penchant for PA, considered as one of the two exposure variables of this study, the investigation recorded the patients’ characteristics related to the prognostic factors resulting from patient and organ survival analyses as reported in the annual CNT-SIT register publications [27]. The following demographic features were collected: donor age; recipient gender and age at the transplant (18–40, 41–50, 51–60, >60 years) and at the interview time (18–49, 50–59, 60–69, ≥70 years), grouped according to the quartile distribution; body mass index (BMI; normal weight, overweight, obese and underweight) [28]. The peri-transplant parameters evaluated were the following: diagnostic indication for transplant, Panel Reactive Antibody (PRA; 0–20; 21–79; ≥80), type of kidney transplant: single, double or combined with other organs (multi-organ kidney transplantation). In addition, other factors included in the analysis were: case-mix (the severity index for patients registered for a renal transplantdefined in terms of clinical complexity and comorbidities), divided into four groups (Standard, Weak, Intermediate and High) and dialysis vintage. The post-transplant factors analyzed were delayed graft function (DGF), defined as need for dialysis in the first 10 days after transplant, and follow-up duration (years) [29,30].

The variation in allograft kidney function, expressed as eGFRwas calculated using the *CKD*-*EPI* formula (mL/min/1.73 m^2^) [31]. The values were computed at the time of discharge after transplant surgery (18–32 days after transplant as baseline) and then yearly at the follow-up visits.

### Statistical analysis

As preliminary analysis, demographic and clinical factors possibly involved in the penchant for PA (outcome variable: active versus non-active) were evaluated. Categorical variables are presented as frequencies and percentages, while continuous variables are given as mean and standard deviations. Chi-square test and adjusted for unequal variance independent sample *t*-tests were performed, as appropriate. The univariate logistic regression analysis for the mentioned factors and the multivariable analysis were implemented.

A subset of patients with at least 10-year follow-up were further evaluated with the aim of assessing the longitudinal changes in graft function, using eGFR values at discharge after transplant (baseline) and at the annual follow-up visits, in the whole group and in KTRs stratified according to age ranges. A longitudinal multivariable linear regression model was applied to determine the relationship between the yearly change in eGFR after transplantation and the performance related to PA [32,33]. The analysis was conducted by fitting a mixed-effect (random-and-fixed) linear-regression model (for repeated measures), considering that the model assumes that the determinations from a single subject share a set of latent, unobserved, random effects which are used to generate an association structure between the repeated measurements [34]. Some other simple theoretical details are presented in supplemental digital content (SDC).

Considering the effect of time as a within-patient factor [34], the model sought to identify the pattern of changes in eGFR over time as post-transplant outcome. For this analysis, the starting point of observation was the eGFR value observed at 1-year from renal transplant, excluding eGFR at discharge from the analysis, as this parameter can present a great variability. Therefore, to avoid the inter-individual differences frequently found in graft function recovery after transplant, the model was targeted on eGFR values at one year after renal transplant. The mixed model finally fitted included in addition to the fixed baseline factors, a random intercept corresponding to the single KTR together with its random slopes consisting of recipient’s age at follow-up visit and the (ordinal) number of follow-up visits.

Estimation of the parameters is based on restricted maximum likelihood (REML). A test for factor interaction was also performed (Wald test, as appropriate). The test statistic of the Likelihood-Ratio test [LR = −2(L1–L0), approximately χ2 distributed] was performed after the model estimation, to select the best fit.

The mixed-effect regression models were fitted to this longitudinal panel after propensity score matching (PSM) being implemented to take into account and limit selection biases between the two groups (active and non-active). By applying PSM that is the probability of receiving a ‘treatment’ conditional on observed covariates, we adjusted for differences of background (baseline) factors [35] (see SDC). Provided that the included variables were thought to be related to both penchant for PA and clinical outcome (eGFR trend over time), PSM was used to reduce confounding factors.

In the automated algorithm used for one-to-one PSM between groups, the variables to be included in the analysis were firstly chosen by running the multivariable logistic regression analysis, allowing the sample divided into balanced quintiles. The patients were evaluated with the caliper set at a 1/4 of standard deviation of the propensity scores. Covariates used to calculate the propensity index, based on previous literature evidence, were: donor age, year of the transplant, patients’ gender, patient age at transplant, PRA groups, dialysis vintage and DGF.

The amount of missing eGFR data (average about 15% from the first annual follow-up) was randomly missing and, therefore, we considered their impact irrelevant on the results, then no imputation method was applied also in consideration of the statistical technique applied (mixed regression analysis) [36].

The 95% confidence intervals are detailed, along with the point estimations; a p value equal or below 0.05 was considered statistically significant. The strengthening the reporting of retrospective observational studies in epidemiology (STROBE Statement) was followed in the manuscript reporting. The analyses were performed using Stata Software, Release 13 (Stata Corp LP, College Station, TX).

### Compliance with ethical standards

This retrospective study was conducted on already available data collected on the basis of written informed consents obtained from all participants in the study. The subjects were completely anonymized and cannot be identified through the article. The CNT is authorized to collect and analyze data by law (law n.91/1999). Furthermore, with the Ministerial Decree n. 130/2019 (DM 20 August 2019, n.130, art. 6, 3), the CNT is authorized to use pseudo-anonymous routine follow-up and transplant center activity data with the aim a deeper knowledge of the patient’s response to transplant also over long time of follow up.

The study follows the principles of the Declaration of Helsinki.

## Results

Figure 1 shows the algorithm used for patients’ selection: out of 22,099 deceased-donor kidney transplants performed in 14 years (2002–2015), 15,239 patients met the eligibility criteria (first kidney transplant at age above 18 years with regular annual follow-up visits) and were interviewed about their PA during the yearly follow-up visits: 6055 (39.7%) KTRs provided their answers, revealing that 3122 (51.6%) were active, and 2933 (48.4%) were non-active. Among them, male were 3122 out of 6055 patients (51.6%), age at transplant was 49.5 ± 12.2 years, the length of follow up period was 6.3 ± 3.9 years, and eGFR value was 60.3 ± 22.4 mL/min/1.73 m^2^.

### The penchant for doing physical activity

Table 1 describes the habits in terms of regular exercise, the baseline clinical and demographic characteristics of the 6055 KTRs, comparing active versus non-active groups. Physically active patients were younger (OR = 0.98 per increasing year, *p* < 0.001), considering both age at the transplant and current age, and that male gender was associated with higher penchant for doing PA (OR = 1.25, 95%CI 1.2–1.4; *p* < 0.001). About the other factors analyzed, normal weight KTRs were the most represented group with the largest proportion of active subjects (1843; 59.0% active versus 1544; 52.6% non-active, respectively), whereas overweight (OR = 0.8, 95%CI 0.7–0.9; *p* < 0.001) and obese patients (OR = 0.5, 95%CI 0.4–0.6; *p* < 0.001) showed a minor penchant for PA. In total, 3.6% of KTRs underwent a multi-organ transplant and this group resulted less prone for performing PA (0.7, 95%CI 1.2–1.4; *p* = 0.002). The subjects with lower dialysis vintage prior to transplantation appeared to be weakly more active, although this finding did not reach statistical significance (OR = 1.2, *p* = 0.3 in the group with 1–2 years of dialysis vintage). Based on the severity index at transplant (case-mix), KTRs with Weak and Intermediate levels presented greater penchant for PA (OR = 1.2, *p* = 0.07, and OR = 1.3, *p* = 0.002, respectively) than those with the Standard level (used as reference group). According to the multivariable logistic regression analysis, the association of annual increase in donors and recipients’ age, as well as the years of follow-up, with lower for the penchant for PA (adjusted-OR of penchant for PA) was confirmed. Likewise, males showed increased penchant for doing PA (OR = 1.3). Overweight or obese patients (OR = 0.84 and OR = 0.48, respectively) and those with longer dialysis vintage (OR = 0.98 every year of dialysis) also showed a link with lower penchant for PA (Table 2).

**Table 1.** Penchant for doing Physical Activity in relation to the clinical and demographic characteristics of the Italian KTR population.

		Penchant for doing physical activity									
		Yes		No		Total					
		3122	*51.6%*	2933	*48.4%*	6055	*100%*	*p*-value^(1)^	OR	*95% IC*	*P*>|z|^(2)^
Donor age	Mean ± SD	47.1 ± 16.8		51.3 ± 17.2		49.1 ± 17.1		*<0.001*	0.986	*0.983–0.989*	*<0.001*
Patient age at transplant	Mean ± SD	47.8 ± 12.4		51.4 ± 11.8		48.5 ± 12.2		*<0.001*	0.9759	*0.9172–0.9879*	*<0.001*
	Age group	*N*	%	*N*	%	*N*	*%*				
	18–40	885	*28.3*	570	*19.4*	1455	24.0	*<0.001*	**1**		
	41–50	878	*28.1*	706	*24.1*	1584	26.2		0.80	*0.69–0.93*	*0.003*
	51–60	859	*27.5*	947	*32.3*	1806	29.8		0.58	*0.51–0.67*	*<0.001*
		500	*16.0*	710	*24.2*	1210	20.0		0.45	*0.39–0.53*	*<0.001*
Patient age at	Mean ± SD	55.0 ± 12.2		58.6 ± 11.8		56.8 ± 12.1		*<0.001*	0.98	*0.97–0.99*	*<0.001*
extraction	Age group	*N*	%	*N*	%	*N*	%				
	18*–*49	986	*31.6*	631	*21.5*	1617	*26.7*	*<0.001*	1		
	50*–*59	896	*28.7*	758	*25.8*	1654	*27.3*		0.76	0.66*–*0.87	*<0.001*
	60*–*69	842	*27.0*	971	*33.1*	1813	*29.9*		0-55	0.48*–*0.64	*<0.001*
	>=70	398	*12.7*	573	*19.5*	971	*16.0*		0.44	0.38*–*0.52	*<0.001*
Years of follow up	Mean ± SD	6.4 ± 4		6.2 ± 3.8		6.3 ± 3.9		*0.04*	1.014	*1.0006–1.027*	*0.04*
		*N*	*%*	*N*	*%*	*N*	*%*				
Gender	F	1054	*33.8*	1143	*39.0*	2197	*36.3*	*<0.001*	1		
	M	2068	*66.2*	1790	*61.0*	3858	*63.7*		1.25	*1.2–1.4*	*<0.001*
Type of kidney transplant	Single	2874	*92.1*	2629	*89.6*	5503	*90.9*	*<0.001*	1		
	Double	156	*5.0*	175	*6.0*	331	*5.5*		0.8	*0.6–1.0*	*0.07*
	Combined	92	*2.9*	129	*4.4*	221	*3.6*		0.7	*0.5–0.9*	*0.002*
BMI	Normal weight	1843	*59.0*	1544	*52.6*	3387	*55.9*	*<0.01*	1		
	Overweight	906	*29.0*	946	*32.3*	1852	*30.6*		0.8	*0.7–0.9*	*<0.001*
	Obese	149	*4.8*	260	*8.9*	409	*6.8*		0.5	*0.4–0.6*	*<0.001*
	Underweight	170	*5.4*	135	*4.6*	305	*5.0*		1.1	*0.8–1.3*	*0.7*
	MD	54	*1.7*	48	*1.6*	102	*1.7*				
PRA	0-20	2837	*90.9*	2647	*90.2*	5484	*90.6*	*0.8*	1		
	21-79	176	*5.6*	173	*5.9*	349	*5.8*		0.9	*0.8–1.1*	*0.6*
	>=80	61	*2.0*	62	*2.1*	123	*2.0*		0.9	*0.7–1.2*	*0.4*
	MD	48	*1.5*	51	*1.7*	99	*1.6*				
Diagnosis	Glomerular nephropathies	1196	*38.3*	1108	*37.8*	2304	*38.1*	*<0.001*	1		
	Diabetic nephropathy	92	*2.9*	172	*5.9*	264	*4.4*		0.5	*0.4–0.6*	*0.001*
	Cystic nephropathies Congenital nephropathies Uropathies	522	*16.7*	554	*18.9*	1076	*17.8*		0.873	*0.76–1.009*	*0.07*
	Hypertensive nephrosclerosis & Nephrovasculopathy	128	*4.1*	168	*5.7*	296	*4.9*		0.8	*0.6–0.97*	*0.012*
	Other kidney diseases ^(3)^	1184	*37.9*	931	*31.7*	2115	*34.9*		1.2	*1.0–1.4*	*0.007*
Dialysis vintage (years)	* Preemptive*^(4)^ or <1	553	*17.7*	504	*17.2*	1057	*17.5*	*<0.009*	1		
	1–2	525	*16.8*	452	*15.4*	977	*16.1*		1.2	*0.9–1.5*	*0.3*
	2–3	499	*16.0*	460	*15.7*	959	*15.8*		1.1	*0.7–1.1*	*0.2*
	3–4	466	*14.9*	402	*13.7*	868	*14.3*		1.0	*0.8–1.2*	*0.6*
	>4	1079	*34.6*	1115	*38.0*	2194	*36.2*		0.942	*0.786–1.13*	*0.5*
Case mix severity index at transplant	Standard	659	*21.1*	685	*23.4*	1344	*22.2*	*<0.0001*	1		
	Weak	860	*27.5*	711	*24.2*	1571	*25.9*		1.2	*1.0–1.3*	*0.07*
	Intermediate	787	*25.2*	714	*24.3*	1501	*24.8*		1.3	*1.1–1.5*	*0.002*
	High	813	*26.0*	821	*28.0*	1634	*27.0*		1.0	*0.9–1.2*	*0.7*
	MD	3		2		5					
DGF	NO	2319	*74.3*	2080	*70.9*	4399	*72.7*	*<0.002*	1		
	YES	801	*25.7*	850	*29.0*	1651	*27.3*		0.846	*0.755–0.9471*	*0.004*

OR: odds ratio; MD: missing data, not included in the univariate logistic regression analysis; BMI: body mass index according to the WHO classification. (1) *p*-value for Pearson χ2 test or *t*-test, as appropriate; (2) OR *p*-value in the univariate logistic regression; (3) other kidney diseases include: 371 Tubulointerstitial nephropathies; 13 renal cancers; 193 acute renal failures and 1426 other kidney diseases; (4) preemptive: no dialysis treatment before transplant.

**Table 2.** Penchant for doing physical activity (factors at the baseline). Multivariable logistic analysis.

	OR	p-value	95%IC
Donor age (years)	0.99	0.01	[0.989–0.997]
Patient age at the transplant (years)	0.98	0.001	[0.972–0.984]
Male versus female	1.30	0.001	[1.167–1.449]
Case mix severity index at transplant			
Weak versus Standard	1.44	0.001	[1.239–1.675]
Intermediate versus Standard	1.61	0.001	[1.373–1.884]
High versus Standard	1.40	0.001	[1.198–1.636]
BMI			
Overweight (25–30) versus Normal weight	0.84	0.01	[0.746–0.940]
Obese (30–70) versus Normal weight	0.48	0.001	[0.387–0.596]
Dialysis vintage			
>4 years versus <=1 year	0.81	0.001	[0.726–0.909]
Follow up from transplant (year)	0.98	0.01	[0.964–0.991]
Observations	6,055		

OR >1: Penchant for perform Physical Activity.

### eGFR: graft function in the whole population

**Figure 2.**
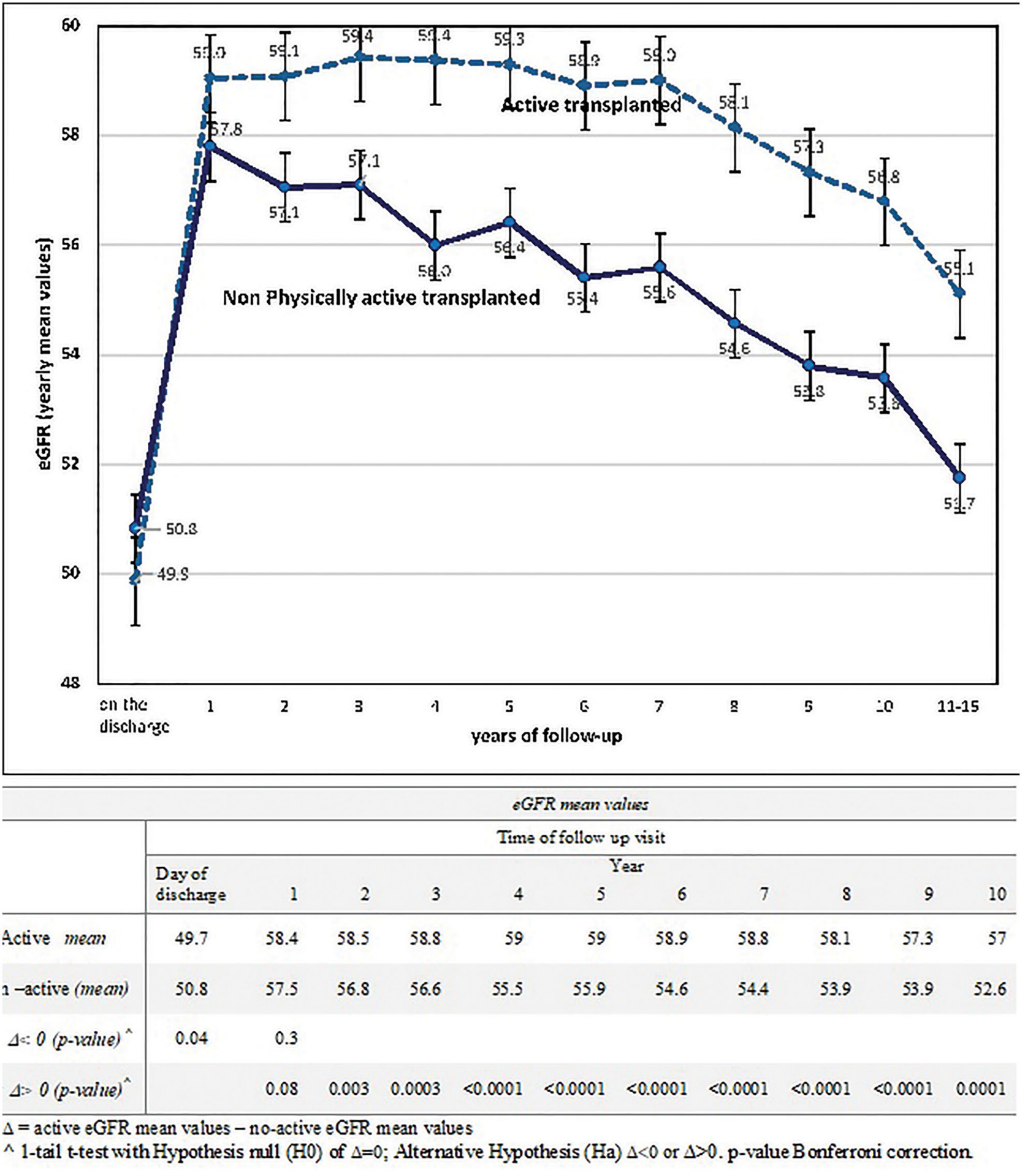
eGFR mean values on kidney transplant discharge and yearly follow-up visit.

We recorded eGFR the values observed starting from the day of discharge after transplant and then at the annual follow-up visits in 6055 KTRs. The means and 95% CI of eGFR active and non-active patient groups, along with their comparisons, are illustrated for each follow-up measurement in Figure 2. In our study, graft function at the discharge time day was lower compared to the values of the first year, and then eGFR values showed a steady decrease in the following years.

**Figure 3.**
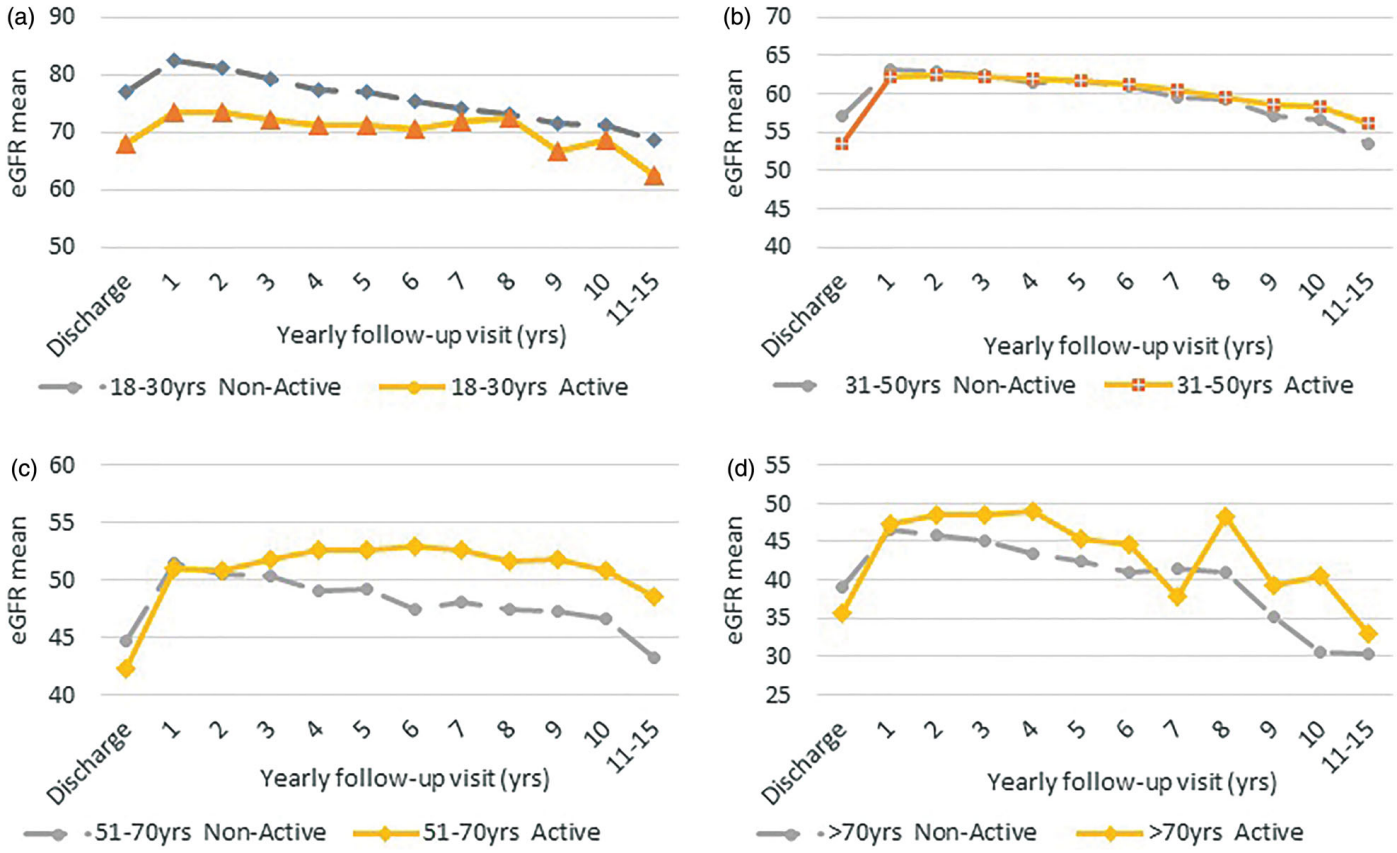
Mean eGFR values trend starting from kidney transplant discharge and 10-annual follow-up visits. Active versus non-active KTRs in all age groups (18–30, 31–50, 51–70, over 70 years).

In the comparison between the groups, starting from the second year of follow-up, active patients showed significantly higher eGFR values (see table in Figure 2). Figure 2 represents the results of the eGFR-mean comparisons between active and non-active KTRs over 10-years of observation for the whole study population. At the time of discharge after transplant active subjects showed lower eGFR values; however, in the successive years, these patients displayed a better long-term preservation of their graft function. After stratifying recipients by age groups (18–30, 31–50, 51–70, over 70 years), eGFR of non-active KTRs was found to be lower compared to that of active patients (Figure 3), particularly in the patients aged more than 50 years.

### Penchant for PA and eGFR levels in the subset of patients with at least 10-year follow-up

The eGFR values of the subset of the 2060 KTRs with at least 10-year follow-up were then analyzed to assess the possible association between the penchant for PA and graft function changes in the long-term. This subgroup of patients presented similar characteristics to those in the whole population of 6055 KTRs, although DGF and dialysis vintage lost statistical significance (see univariate analysis in Table S2.1). In the multivariable analysis, male subjects showed higher OR of penchant for doing PA than women (adjusted-OR = 1.28; 95%CI 1.07–1.54). With increasing donors and recipients’ age, a reduced penchant for doing PA was observed (adjusted-OR decreased by 1% with the increase of each year of age in the donors and 2% in KTRs, respectively). Compared to the lower PRA value (reference category), intermediate levels (between 20 and 79) showed a lower penchant for doing PA (adjusted-OR = 0.69; 95%CI 0.49–1.05; *p* = 0.08). Patients with mild or intermediate case mix severity index displayed a higher tendency for performing PA (adjusted-OR = 1.42 and adjusted-OR = 1.45, respectively) compared to those with standard case mix (Table 3).

**Table 3.** Multivariable analysis to detect the penchant for doing physical activity factors in the subset of 2060 KTRs with 10-years of follow up or more.

Outcome: physical activity y/n	OR	P > z	95 %Conf	Interval
Donor age (year)	0.99	0.001	0.98	1.00
Age patient (at the KT time)	0.98	<0.001	0.97	0.99
Sex: male VERSUS female	1.28	0.01	1.07	1.54
Case mix:				
Weak VERSUs Standard	1.42	0.001	1.15	1.75
Intermediate VERSUS Standard	1.45	0.001	1.15	1.82
PRA: 21–79 VERSUS 0–20	0.69	0.08	0.46	1.05
*Observations*	2,060			

### The eGFR trajectories over time in the subset of patients

To analyze the changes of eGFR values over time, starting from 1-year after kidney transplant (when the recovery of allograft function can be considered complete), given that multiple eGFR measures from the same KTR are more similar than those from other KTRs, a mixed-effect linear regression model which contained the eGFR values on the follow-up visit as random effect was fit to the data in a step-wise procedure. Beginning from the simple (random intercept-only) model which allows for KTR (considered as cluster) effects on eGFR values over time and without explanatory variables, the between-KTR variance in eGFR values was estimated as s^2^ = 336.1, and the within-KTR variance was estimated as s_e_^2^=108.9 (with Likelihood-Ratio test, LR test, versus linear regression: 18,734.11; χ2-*p* < 0.001). The individual trajectories of the eGFR values occurred during the years of follow-up were then analyzed introducing the random slope factor identified as the follow-up-visit-number along with the KTR at the visit (being likewise included as fixed variables): the values of the random coefficient variance were s^2^_time_ = 3.76 and s^2^_KTRage_ = 0.22 (for a detail note, we specify that both LR tests assessed in the random slopes step-wise models with firstly the inclusion of the follow-up-visit-number and subsequently also of KTR age determined χ2(3)-*p* < 0.001, allowing the choice in favor of the more complete model). By including the statistically significant fixed-factors, the final minimal adequate model performed significantly better to an intercept-random-only base-line model (LR test – χ2(1), *p* < 0.0001). Results are shown in Table 4. Both the occurrence of DGF (*b*=-5.5; 95%CI −6.96; −4.0; *p* = 0.004) and female gender (*b*= −1.76; 95%CI −3.1; −0.39; *p* = 0.012) presented very strong negative coefficients. The other coefficients were patient at the follow-up visit (*b*= −0.1 per year; 95%CI −0.18; -0.93 *p* = 0.007), donor age (*b*= −0.50 per year; 95%CI −0.56; −0.45; *p* < 0.001), and the (ordinal) number of the follow-up visit (*b*= −0.62; 95%CI −0.77; −0.46; *p* < 0.001) (Table 4). Surprisingly, diabetic nephropathy diagnosis before kidney transplant was associated with a better transplant outcome (coefficient *b* = 5.1; 95%CI 1.6; 8.7; *p* < 0.001) (Table 4), but this population has a very limited size (3.8% KTRs, Table S2.1).

**Table 4.** Multivariate mixed regression analysis for the eGFR trend and Propensity Score Match adjustment.

Mixed regression analysis	Coefficient (b)	*Robust standard error*	*p*>|Z|	95%Conf.	Interval
Fixed effect parameters					
Physical activity × follow up-visit	0.32	*0.081*	*<0.001*	0.16	0.48
Follow up-visit	−0.62	*0.077*	*<0.001*	−0.77	−046
Donor age (years)	−0.50	*0.027*	*<0.001*	−0.56	−0.45
Patient age at follow up (years)	−0.1	*0.038*	*0.007*	−0.18	−0.03
Female versus male	−1.76	*0.07*	*0.012*	−3.1	−0.39
Diabetic nephropathy	5.1	*1.8*	*0.004*	1.6	8.7
Delayed graft function: yes versus no	−5.5	*0.74*	*<0.001*	−6.96	−4.00
Intercept	*85.4*	*1.03*	*<0.001*	*82.9*	*88.1*
Random-effects parameters^a^					
Variance of random-slopes					
Var (follow up-visit)^b^	3.89	0.24		3.44	4.39
Var (KTR’s age)	0.05	0.06		0.01	0.43
Variance of random-intercept					
Var (between-KTRs)	268.88	12.72		245.07	295.01
Covariance:					
Cov (follow up-visit. KTR age)	−0.26	0.08		−0.42	−0.09
Cov (follow up-visit. KTR)	−11.67	1.25		−14.12	−9.23
Cov (KTR age. KTR)	−0.99	0.42		−1.81	−0.18
Var (within-KTR)	61.57	2.02		57.75	65.65
After Propensity Score match					
Physical Activity × time	0.39	*0.09*	*<0.001*	0.21	0.56
Time	−0.75	*0.06*	*0.039*	−0.88	−0.63

Var: variance; Cov: covariance. *Note*: for active and non-active coefficients we present values of coefficients × visit. Estimation of the parameters is based on restricted maximum likelihood (REML).

^a^Variances and covariances of the random effects (intercept and random slopes) have been reported for additional information on the model fit and to provide detailed technical specifications.

^b^Follow up visit is the ordinal number of visits occurred in the considered study period till no more 10 years, therefore the values come from 1 to 10 (baseline visit excluded).

Finally, the PA (considered in interaction with the follow-up time, *p* < 0.001 in the Wald-test for interaction), in the comparison with the non-PA (reference group) resulted positively associated with the eGFR trend (adjusted coefficient *b* = 0.31; 95%CI 0.16; 0.48; *p* < 0.001), revealing a contrasting effect against the eGFR decline over years (Table 4). In the table, variance and covariances of the factors in the model are also presented.

This mixed model was therefore performed also using Propensity-Score-Match (PSM) approach: the PSM algorithm was obtained from the balancing process by grouping based on the confounding factors associated to the penchant to PA resulting from the preliminary matching analysis.

The factors selected (dummy variables) were: gender, diagnostic indications for transplant (diabetic nephropathy), BMI (obese), patients’ groups of age (35–50, 51–60 and over 60 years), donor groups of age (51–60 and over 60 years), PRA (21–79), time on dialysis prior to transplantation (more than 4 years). At the end of the process, the PSM-algorithm succeeded in satisfying the property of balancing the one-to-one matching for 2052 records, while 8 cases (one active and seven non-active, respectively) were dropped due to impossibility of matching. The 4 balanced blocks resulted homogeneous according to the (propensity) score and confirmed that the mean propensity score was not different for active and non-active subjects in each block, thus, allowing a stratified mixed regression analysis.

The results of the final PSM-adjusted analysis kept pointing an analogous coefficient value of the activity (*b* = 0.39; 95%CI 0.21; 0.56, *p* < 0.001; non-active reference; bottom of Table 4).

**Figure 4.**
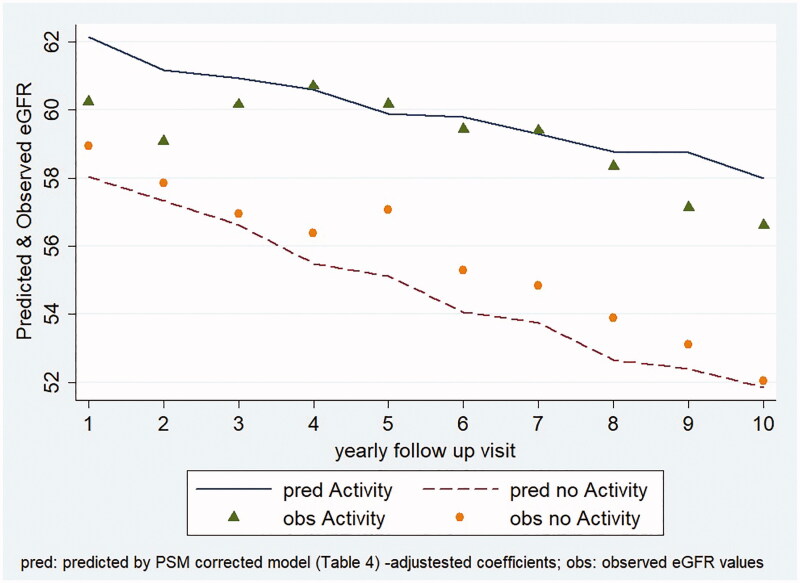
eGFR trend predicted by multivariable regression mixed model versus observed mean values.

Lastly, based on the after PSM adjusted coefficients from the mixed regression model, the adjusted predictive plot (Figure 4), showing the appropriateness of the model fit, further confirms the effects of PA on e-GFR trend: the active KTRs, also after adjustment for the significant factors, confirmed the slower decline of their graft function.

## Discussion

To the best of our knowledge, this is the first large retrospective observational study that investigates the penchant for regular PA in KTR population and its association with renal function in the long-term. Our analysis collected and processed all the compulsorily records for all patients in the Italian registry (SIT), and therefore, it is representative of the entire Italian KTR population; these data are not reported in any other national register.

In the study cohort related to available lifestyle information (active, non-active), a mere 51.6% reaches the levels of PA recommended by the WHO international guidelines, in line with previous data from other authors for CKD patients and transplant recipients [16,19,37,38].

In the Italian KTR population, as expected, there is a relative greater penchant for PA in younger patients; and this tendency decreases progressively with increasing age (after 50-years the sedentary behavior prevails). Our data on the habits of the Italian general population are consistent with the few currently available studies focused on PA and age in transplanted patients [39,40]. Moreover, as the duration of the dialysis prior to kidney transplantation increases, the penchant for doing PA reduces progressively. The population on dialysis treatment shows a high prevalence of sedentary behaviors [41], and this is particularly true for the elderly patients. Progressive fitness decay, development of cardiovascular and osteoarticular comorbidities [42] with negative impact on psychological aspects contribute to explain the results. Furthermore, overweight and obese patients are less prone to perform PA. Our results are in line with the study of Kang et al. who recorded a more intense PA among younger male subjects, with a small proportion of overweight or obese subjects [43]. In our study, women demonstrated a lower inclination for physical exercise. However, this finding may be affected by social context and educational level [39,44] of the Italian population and further investigations are necessary to highlight the detailed reasons underlying this trend. Overall, the results show a population needing a stronger motivation to maintain good adherence to correct lifestyles while getting older, maybe due to the increased fragility.

This retrospective study contributes to confirm the positive long-term role of PA on graft function preservation in the transplanted population on a large scale, as it has been already reported in the general population and for chronic diseases [45]. Possible mechanisms include: reduction of blood pressure, improvement of glycemic control, an increase in nitric oxide production, upregulation of endothelial nitric oxide production, reduction of inflammatory status. Indeed, with regard to renal function, active KTRs showed higher eGFR than non-active KTRs. In particular, in the group within the age range 51–70 years, graft function revealed the highest benefits from PA: at this age, chronic degenerative diseases arise more frequently and, on the other hand, the positive effect of the exercise is well documented [46–48].

The overall results of our study are confirmed by the analysis of a subset of patients with at least 10-year follow-up observation: multivariable mixed regression displayed a tendency over time to a better preservation of graft function in patients who declared to practice regular PA. Since the CNT-SIT is a national registry, such organized system collects a great amount of homogeneous data, to evaluate specific outcomes for a defined population, with predetermined scientific-clinical-policy purposes. Consequently, the methodological effort in the development of data analysis was aimed at identifying and counteracting potentially confounding factors deriving from patients' conditions and different comorbidities.

Some authors describe better transplant outcomes in active patients [16,49–52]; in several studies a different trend is reported compared to non-active patients [53–56]. The most recent reviews are not able to draw firm conclusions about the efficacy of the PA on renal function progress [10,57,59], although the available studies do not reach long-term follow-up. The positive effects of active lifestyle on graft function preservation might have a better observable impact in the long-term. It is well-assessed that the factors involved in the progression of organ damage and in the general outcome of the graft are manifold, thus research efforts should be focused on the modifiable ones, such as lifestyle. A recent review in non-dialysis CKD and renal transplant recipients related to survival rates was correlated with greater PA and physical function levels [58].

In our study, multivariable analysis highlights the importance of the adherence to the active lifestyle for the preservation of renal function, also KTRs. The results confirm the experience gained so far in ‘Transplant… and now it’s time for sport’, a program started in 2008 and sponsored by CNT which, with the collaboration of the patients’ association ANED (National Association of Dialysis Hemodialysis and Transplant), that has strongly promoted the implementation of post-transplant PA [59].

This study presents strengths and limitations. Strengths include the large sample size of the entire Italian kidney transplant population, the long-term period of follow-up, and the application of analysis techniques of repeated observations. Our investigation was firstly addressed to identify potential relationships between PA and renal function. To minimize the risk of bias, the analysis relied on the entire population of transplant recipients, and then applied specific statistical analyses (mixed effects regression for repeated measures along with propensity score adjustment) on a restricted subset of long-term follow-up patients. The main findings allow to confirm the positive effects of PA practice in this fragile population, and also after adjustment for many important confounding factors, the comparison of active versus non-active groups revealed a substantial difference in terms the functional decline of the graft. However, some limits deserve to be mentioned. First, only surviving KTRs and with functioning graft were analyzed: KTRs who dropped out of follow-up due to acute events (death, graft loss) or discontinued visits were not evaluated. Moreover, the KTRs were interviewed only once (one-time-observation), independently from their having an active lifestyle in another period of their lives, and no further information was gathered on the details of the exercise performed; no data were available about PA levels previous to kidney transplant surgery. Lastly, data relating to the dysmetabolic changes in post-transplant period are lacking.

For the transplant community, these findings can be a spur to elaborate further interventions on sedentary lifestyle before and after transplantation [51,52] and lead us to ponder about how to overcome barriers to PA [31,53–56] in the most fragile patients (elderly, long-term dialysis). The lack of specific counseling by physicians about the benefits of PA could be a critical issue, and recommendations alone may be not sufficient to induce actual lifestyle changes [59–67]. Development of regional and national networks with multidisciplinary teams may constitute an important starting point to promote the spread of territorial programs of PA (supervised or unsupervised exercise) [20,60–67]; the availability of the national registry represents an added value for verify and monitor their efficacy.

In conclusion, this study suggests that in the Italian KTR population, the prevalence of non-active subjects is high; this tendency grows with age and with the duration of dialysis treatment prior to transplantation; a lower PA is observed in overweight and obese patients.

Physical inactivity is a modifiable factor for prevention of both cardiovascular risk and progression of a graft damage. In our population of transplant recipients, eGFR values were found to be higher in active than in sedentary patients, and this difference was more pronounced in the elderly. Renal function data were confirmed in the population with a follow-up of at least 10 years, and better transplant outcomes were observed in the active group. In a population with a high cardiovascular risk, such as KTRs, it is advisable to include PA as a non-pharmacological therapy to improve long-term results in terms of morbidity, mortality and graft survival.

Prospective and interventional studies would help to confirm our results. Future strategies for further implementations of PA should get the attention of professional care providers and institutions that can assess the impact and the sustainability of programs to promote and guide patients toward a healthier lifestyle.

## Supplementary Material

Supplemental Material

Supplemental Material

## References

[1] Cannon CP. Cardiovascular disease and modifiable cardiometabolic risk factors. Clin Cornerstone. 2007;8(3):11–28. 18452839 10.1016/s1098-3597(07)80025-1

[2] Heiwe S, Jacobson SH. Exercise training in adults with CKD: a systematic review and meta-analysis. Am J Kidney Dis. 2014;64(3):383–393. 24913219 10.1053/j.ajkd.2014.03.020

[3] Barcellos FC, Santos IS, Umpierre D, et al. Effects of exercise in the whole spectrum of chronic kidney disease: a systematic review. Clin Kidney J. 2015;8(6):753–765. 26613036 10.1093/ckj/sfv099 PMC4655802

[4] Zelle DM, Klaassen G, van Adrichem E, et al. Physical inactivity: a risk factor and target for intervention in renal care. Nat Rev Nephrol. 2017;13(5):318. 28405009 10.1038/nrneph.2017.44

[5] Greenwood SA, Koufaki P, Mercer TH, et al. Effect of exercise training on estimated gfr, vascular health, and cardiorespiratory fitness in patients with CKD: a pilot randomized controlled trial. Am J Kidney Dis. 2015;65(3):425–434. 25236582 10.1053/j.ajkd.2014.07.015

[6] Obermayr RP, Temml C, Knechtelsdorfer M, et al. Predictors of new-onset decline in kidney function in a general middle-european population. Nephrol Dial Transplant. 2008;23(4):1265–1273. 18039642 10.1093/ndt/gfm790

[7] Robinson-Cohen C, Katz R, Mozaffarian D, et al. Physical activity and rapid decline in kidney function among older adults. Arch Intern Med. 2009;169(22):2116–2123. 20008696 10.1001/archinternmed.2009.438 PMC2878734

[8] Zelle DM, Corpeleijn E, Stolk RP, et al. Low physical activity and risk of cardiovascular and all-cause mortality in renal transplant recipients. CJASN. 2011;6(4):898–905. 21372213 10.2215/CJN.03340410 PMC3069385

[9] Li C, Xu J, Qin W, et al. Meta-analysis of the effects of exercise training on markers of metabolic syndrome in solid organ transplant recipients. Prog Transplant. 2018;28(3):278–287. 29898634 10.1177/1526924818781576

[10] Didsbury M, McGee RG, Tong A, et al. Exercise training in solid organ transplant recipients: a systematic review and meta-analysis. Transplantation. 2013;95(5):679–687. 23364480 10.1097/TP.0b013e31827a3d3e

[11] Macdonald JH, Kirkman D, Jibani M. Kidney transplantation: a systematic review of interventional and observational studies of physical activity on intermediate outcomes. Adv Chronic Kidney Dis. 2009;16(6):482–500. 19801137 10.1053/j.ackd.2009.07.011

[12] Clayton PA, Lim WH, Wong G, et al. Relationship between eGFR decline and hard outcomes after kidney transplants. J Am Soc Nephrol. 2016;27(11):3440–3446. 27059513 10.1681/ASN.2015050524 PMC5084878

[13] Henry RM, Kostense PJ, Bos G, et al. Mild renal insufficiency is associated with increased cardiovascular mortality: the Hoorn study. Kidney Int. 2002;62(4):1402–1407. 12234312 10.1111/j.1523-1755.2002.kid571.x

[14] Stump CS. Physical activity in the prevention of chronic kidney disease. Cardiorenal Med. 2011;1(3):164–173. 22258539 10.1159/000329929 PMC3150958

[15] Totti V, Fernhall B, Di Michele R, et al. Longitudinal analysis of cardiovascular risk factors in active and sedentary kidney transplant recipients. Medicina (Kaunas). 2020;56(4):183. 10.3390/medicina56040183 PMC7230877 32316125

[16] Gordon EJ, Prohaska TR, Gallant MP, et al. Longitudinal analysis of physical activity, fluid intake, and graft function among kidney transplant recipients. Transplant Int. 2009;22(10):990–998. 10.1111/j.1432-2277.2009.00917.x PMC2925536 19619168

[17] Svensson M, Jardine A, Fellström B, et al. Prevention of cardiovascular disease after renal transplantation. Curr Opin Organ Transplant. 2012;17(4):393–400. 22790074 10.1097/MOT.0b013e3283560a3b

[18] Bellizzi V, Cupisti A, Capitanini A, et al. Physical activity and renal transplantation. Kidney Blood Press Res. 2014;39(2–3):212–219. 25118089 10.1159/000355799

[19] Zelle DM, Kok T, Dontje ML, et al. The role of diet and physical activity in post-transplant weight gain after renal transplantation. Clin Transplant. 2013;27(4):E484–E490. 23758229 10.1111/ctr.12149

[20] Roi GS, Mosconi G, Totti V, et al. Renal function and physical fitness after 12-mo supervised training in kidney transplant recipients. World J Transplant. 2018;8(1):13–22. 29507858 10.5500/wjt.v8.i1.13 PMC5829451

[21] Ikizler TA, Robinson-Cohen C, Ellis C, et al. Metabolic effects of diet and exercise in patients with moderate to severe CKD: a randomized clinical trial. J Am Soc Nephrol. 2018;29(1):250–259. 29038285 10.1681/ASN.2017010020 PMC5748901

[22] Kohler M, Schanzer W, Thevis M. Effects of exercise on the urinary proteome. Adv Exp Med Biol. 2015;845:121–131. 25355575 10.1007/978-94-017-9523-4_12

[23] Poortmans J, Labilloy D. The influence of work intensity on postexercise proteinuria. Eur J Appl Physiol Occup Physiol. 1988;57(2):260–263. 3349996 10.1007/BF00640673

[24] Mosconi G, Roi GS, Totti V, et al. Renal function in kidney and liver transplant recipients after a 130-km road cycling race. Transplant Direct. 2015;1(9):e36. 27500236 10.1097/TXD.0000000000000546 PMC4946485

[25] https://trapianti.sanita.it/statistiche/home.aspx. Updated April 1999. Accessed April 7, 2017.

[26] KDIGO clinical practice guideline for the care of kidney transplant recipients. Kidney Disease: Improving Global Outcomes (KDIGO) Transplant Work Group. Am J Transplant. 2009;9(Suppl 3):S1–S155. 10.1111/j.1600-6143.2009.02834.x 19845597

[27] https://trapianti.sanita.it/statistiche/valutazione_attivita.aspx. Updated April 1999. Accessed April 7, 2017.

[28] WHO BMI classification: http://www.euro.who.int/en/health-topics/disease-prevention/nutrition/a-healthy-lifestyle/body-mass-index-bmi.

[29] Ravanan R, Udayaraj U, Ansell D, et al. Variation between centres in access to renal transplantation in UK: longitudinal cohort study. BMJ. 2010;341(1):c3451. 20647283 10.1136/bmj.c3451 PMC2907479

[30] Oniscu GC, Schalkwijk AAH, Johnson RJ, et al. Equity of access to renal transplant waiting list and renal transplantation in Scotland: cohort study. BMJ. 2003;327(7426):1261. 14644969 10.1136/bmj.327.7426.1261 PMC286245

[31] Levey S, Stevens LA. Estimating GFR using the CKD Epidemiology Collaboration (CKD-EPI) creatinine equation: more accurate GFR estimates, lower CKD prevalence estimates, and better risk predictions. Am J Kidney Dis. 2010;55(4):622–627. 20338463 10.1053/j.ajkd.2010.02.337 PMC2846308

[32] Vigil A, Condés E, Camacho R, et al. Predictors of a rapid decline of renal function in patients with chronic kidney disease referred to a nephrology outpatient clinic: a longitudinal study. Adv Nephrol Article ID 657624. 2015;2015:1–8.

[33] Marcén R, Morales JM, Fernández-Rodriguez A, et al. Long-term graft function changes in kidney transplant recipients. NDT Plus. 2010;3(Suppl_2):ii2–ii8. 20508857 10.1093/ndtplus/sfq063 PMC2875040

[34] Blackwell E, Mendes de Leon CF, Miller GE. Applying mixed regression models to the analysis of repeated-measures data in psychosomatic medicine. Psychosom Med. 2006;68(6):870–878. 17079709 10.1097/01.psy.0000239144.91689.ca

[35] Joffe MM, Rosenbaum PR. Invited commentary: propensity scores. Am J Epidemiol. 1999;150(4):327–333. 10453808 10.1093/oxfordjournals.aje.a010011

[36] Twisk J, de Boer M, de Vente W, et al. Multiple imputation of missing values was not necessary before performing a longitudinal mixed-model analysis. J Clin Epidemiol. 2013;66(9):1022–1028. 23790725 10.1016/j.jclinepi.2013.03.017

[37] Dontje ML, de Greef MH, Krijnen WP, et al. Longitudinal measurement of physical activity following kidney transplantation. Clin Transplant. 2014;28(4):394–402. 24635476 10.1111/ctr.12325

[38] Regolisti G, Maggiore U, Sabatino A, et al.; on behalf of the Gruppo di Studio “Esercizio fisico nel paziente con insufficienza renale cronica” of the Società Italiana di Nefrologia. Interaction of healthcare staff's attitude with barriers to physical activity in hemodialysis patients: a quantitative assessment. PLoS One. 2018;13(6):e0198987. 29924872 10.1371/journal.pone.0198987 PMC6010259

[39] Cislaghi B, Cislaghi C. Self-rated health as a valid indicator for health-equity analyses: evidence from the Italian health interview survey. BMC Public Health. 2019;19(1):533. 31072306 10.1186/s12889-019-6839-5 PMC6509759

[40] Puggina A, Aleksovska K, Buck C, DEDIPAC Consortium, et al. Policy determinants of physical activity across the life course: a 'DEDIPAC' umbrella systematic literature review. Eur J Public Health. 2017;28(1):105–118. 10.1093/eurpub/ckx174 PMC5881728 29048468

[41] Johansen KL, Chertow GM, Ng AV, et al. Physical activity levels in patients on hemodialysis and healthy sedentary controls. Kidney Int. 2000;57(6):2564–2570. 10844626 10.1046/j.1523-1755.2000.00116.x

[42] Yanishi M, Kinoshita H, Tsukaguchi H, et al. Factors related to osteosarcopenia in kidney transplant recipients. Transplant Proc. 2018;50(10):3371–3375. 30586836 10.1016/j.transproceed.2018.04.032

[43] Kang AW, Garber CE, Eaton CB, et al. Physical activity and cardiovascular risk among kidney transplant patients. Med Sci Sports Exerc. 2019;51(6):1154–1161. 30629045 10.1249/MSS.0000000000001886 PMC6522300

[44] Balducci S, D'Errico V, Haxhi J, Italian Diabetes and Exercise Study 2 (IDES­2) Investigators, et al. Effect of a behavioral intervention strategy for adoption and maintenance of a physically active lifestyle: the Italian diabetes and exercise study 2 (ides_2): a randomized controlled trial. Diabetes Care. 2017;40(11):1444–1452. 28821576 10.2337/dc17-0594

[45] Perez-Terzic CM. Exercise in cardiovascular diseases. Pm R. 2012;4(11):867–873. 23174552 10.1016/j.pmrj.2012.10.003

[46] Rosas SE, Reese PP, Huan Y, et al. Pretransplant physical activity predicts all-cause mortality in kidney transplant recipients. Am J Nephrol. 2012;35(1):17–23. 22156548 10.1159/000334732 PMC3251242

[47] Hartmann EL, Kitzman D, Rocco M, et al. Physical function in older candidates for renal transplantation: an impaired population. Clin J Am Soc Nephrol. 2009;4(3):588–594. 19261824 10.2215/CJN.03860808 PMC2653669

[48] Painter P, Roshanravan B. The association of physical activity and physical function with clinical outcomes in adults with chronic kidney disease. Curr Opin Nephrol Hypertens. 2013;22(6):615–623. 24100215 10.1097/MNH.0b013e328365b43a

[49] Calella P, Hernández-Sánchez S, Garofalo C, et al. Exercise training in kidney transplant recipients: a systematic review. J Nephrol. 2019;32(4):567–579. 30649716 10.1007/s40620-019-00583-5

[50] Teplan V, Mahrova A, Piťha J, et al. Early exercise training after renal transplantation and asymmetric dimethylarginine: the effect of obesity. Kidney Blood Press Res. 2014;39(4):289–298. 25196348 10.1159/000355806

[51] Juskowa J, Lewandowska M, Bartłomiejczyk I, et al. Physical rehabilitation and risk of atherosclerosis after successful kidney transplantation. Transplant Proc. 2006;38(1):157–160. 16504691 10.1016/j.transproceed.2005.12.077

[52] Korabiewska L, Lewandowska M, Juskowa J, et al. Need for rehabilitation in renal replacement therapy involving allogeneic kidney transplantation. Transplant Proc. 2007;39(9):2776–2777. 18021985 10.1016/j.transproceed.2007.08.082

[53] Lima PS, Campos AS, Correa CS, et al. Effects of chronic physical activity on glomerular filtration rate, creatinine, and the markers of anemia of kidney transplantation patients. Transplant Proc. 2018;50(3):746–749. 29661428 10.1016/j.transproceed.2018.02.009

[54] Tzvetanov I, West-Thielke P, D'Amico G, et al. A novel and personalized rehabilitation. program for obese kidney transplant recipients. Transplant Proc. 2014;46(10):3431–3437. 25498067 10.1016/j.transproceed.2014.05.085

[55] Roi GS, Stefoni S, Mosconi G, et al. Physical activity in solid organ transplant recipients: organizational aspects and preliminary results of the Italian project. Transplant Proc. 2014;46(7):2345–2349. 25242784 10.1016/j.transproceed.2014.07.055

[56] Moraes Dias CJ, Anaisse Azoubel LM, Araújo Costa H, et al. Autonomic modulation analysis in active and sedentary kidney transplanted recipients. Clin Exp Pharmacol Physiol. 2015;42(12):1239–1244. 26284458 10.1111/1440-1681.12481

[57] Oguchi H, Tsujita M, Yazawa M, et al. The efficacy of exercise training in kidney transplant recipients: a meta-analysis and systematic review. Clin Exp Nephrol. 2019;23(2):275–284. 30168049 10.1007/s10157-018-1633-8

[58] MacKinnon HJ, Wilkinson TJ, Clarke AL, et al. The association of physical function and physical activity with all-cause mortality and adverse clinical outcomes in nondialysis chronic kidney disease: a systematic review. Ther Adv Chronic Dis. 2018;9(11):209–226. 30364521 10.1177/2040622318785575 PMC6196637

[59] Totti V, Campione T, Mosconi G, et al. The promotion of pre- and post-transplant physical exercise in the Emilia-Romagna region: the network of the program "transplantation, physical activity, and sport". Transplant Proc. 2019;51(9):2902–2905. 31606183 10.1016/j.transproceed.2019.02.074

[60] Totti V, Campione T, Mosconi G, et al. Observational retrospective study on patient lifestyle in the pretransplantation and post-transplantation period in the Emilia-Romagna region. Transplant Proc; S0041. 2020;1345(20):30245–30247. 10.1016/j.transproceed.2020.03.015 32402457

[61] Mathur S, Janaudis-Ferreira T, Wickerson L, et al. Meeting report: consensus recommendations for a research agenda in exercise in solid organ transplantation. Am J Transplant. 2014;14(10):2235–2245. 25135579 10.1111/ajt.12874

[62] Klaassen G, Zelle DM, Navis GJ, et al. Lifestyle intervention to improve quality of life and prevent weight gain after renal transplantation: design of the Active Care after Transplantation (ACT) randomized controlled trial. BMC Nephrol. 2017;18(1):296. 28915863 10.1186/s12882-017-0709-0 PMC5599936

[63] Takahashi A, Hu SL, Bostom A. Physical activity in kidney transplant recipients: A review. Am J Kidney Dis. 2018;72(3):433–443. 29482935 10.1053/j.ajkd.2017.12.005

[64] Chen G, Gao L, Li X. Effects of exercise training on cardiovascular risk factors in kidney transplant recipients: a systematic review and meta-analysis. Ren Fail. 2019;41(1):408–418. 31106657 10.1080/0886022X.2019.1611602 PMC6534232

[65] van Adrichem EJ, Dekker R, Krijnen WP, et al. Physical activity, sedentary time, and associated factors in recipients of solid-organ transplantation. Phys Ther. 2018;98(8):646–657. 29757444 10.1093/ptj/pzy055

[66] Gustaw T, Schoo E, Barbalinardo C, et al. Physical activity in solid organ transplant recipients: participation, predictors, barriers, and facilitators. Clin Transplant. 2017;31(4):e12929. 10.1111/ctr.12929 28185297

[67] Schoo E, Gustaw T, Barbalinardo C, et al. Solid organ transplant recipients' opinions of pre- and post-transplant supervised exercise programmes: a brief report. Physiother Can. 2017;69(2):178–183. 28539698 10.3138/ptc.2016-18EP PMC5435398

