# Supplementary material for: Physical activity and renal function in the Italian kidney transplant population
Source: Ren Fail. 2020 Nov 30;42(1):1192–204. doi: 10.1080/0886022X.2020.1847723 (PMC7717861; doi:10.1080/0886022X.2020.1847723)
Supplement: Supplemental Material [file IRNF_A_1847723_SM8760.docx]

**Supplementary file 1**

For the analysis of response profiles, the class of models known as linear mixed-effects models [S1] allows the characterization and comparison of changes in the outcome of interest over time, as well as the management of incomplete data. Linear mixed-effects models can also handle unbalanced data and modelling covariance in a parsimonious approach.

In the linear mixed-effects models, individuals in the population are assumed to have their own subject-specific mean response trajectories over time: each subject has his/her own specific curve that illustrates longitudinal change in the response variable. The mixed model contains both fixed effects (achieved by modelling the mean response as a characteristic combination assumed shared by all individuals and fixed over time such as baseline characteristics), as well as random effects that are subject-specific effects (unique for a particular individual). The underlying idea is that the data are hierarchically structured and are modelled by introducing random coefficients, constant within a given level but changing across levels. The introduction of random effects provides a flexible way to model the variability and correlation among repeated measures. When the model includes both random intercepts and slopes (or randomly varying coefficients for any functions of time), the variability of the response can change as a function of the times of measurement, and the magnitudes of the correlations between measurements from the same subject can depend on the time between them.

The observed pattern of decline in our patients was relatively constant over the duration of follow up; consequently, we can approximate the pattern of change using linear trends [S2]. In this work, we were interested in quantifying pattern of changes over time of transplant outcome: the model was built to control the patient variability represented by the random intercept of the regression model; the random slopes were then identified by the (ordinal) number of the follow-up and the patient age at the visits.

**Supplementary references.**

**S1.** Peter C. Austin (2011) An introduction to propensity score methods for reducing the effects of confounding in observational studies. *Multivariate Behavioral Research*, 46:3, 399-424.

**S2.** Wiebe, I. W. Gibson, T. D. Blydt-Hansen, D et al (2015) Nickerson rates and determinants of progression to graft failure in kidney allograft recipients with de novo donor-specific antibody. *American Journal of Transplantation*; 15: 2921–2930.
