# Supplementary material for: Physical activity and renal function in the Italian kidney transplant population
Source: Ren Fail. 2020 Nov 30;42(1):1192–204. doi: 10.1080/0886022X.2020.1847723 (PMC7717861; doi:10.1080/0886022X.2020.1847723)
Supplement: Supplemental Material [file IRNF_A_1847723_SM8753.docx]

**Table S2.1.** Penchant for physical activity in the 2,060-patient subgroup with at list 10-year follow-up.

|  | | **Penchant for physical activity** | | | | | | | | | | | | |
| --- | --- | --- | --- | --- | --- | --- | --- | --- | --- | --- | --- | --- | --- | --- |
|  |  | YES | | | NO | | | TOTAL | | *p-value* | OR | *95% IC* | P>\|z\| ^(2)^ |  |
|  |  | N | % | | N | % | N | | % |  | | | |  |
|  |  | 1084 | *52.6%* | | 976 | *47.4%* | 2060 | | *100.0%* |  |  |  |  |  |
| Donor age | Mean +/- SD | 42.7 +/- 16.8 | | | 48.4 +/- 17.1 | | | 45.4 +/- 17.2 | | *<0.001^(1)^* | 0.98 | *0.976-0.986* | *<0.001* |  |
| Patient age at  transplant | Mean +/- SD | 44.8 +/- 11.8 | | | 49.1 +/- 11.5 | | | 46.8 +/- 11.9 | | *<0.001^(1)^* | 0.97 | *0.96-098* | *<0.001* |  |
|  | Age group | N | *%* | | N | % | N | | % | *<0.001* |  |  |  |  |
|  | 18-40 | 402 | *37.1* | | 234 | *24.0* | 636 | | *30.9* |  | 1 |  |  |  |
|  | 41-50 | 317 | *29.2* | | 264 | *27.0* | 581 | | *28.2* |  | 0.7 | *0.6-09* | *0.002* |  |
|  | 51-60 | 262 | *24.2* | | 311 | *31.9* | 573 | | *27.8* |  | 0.5 | *0.4-0.6* | *<0.001* |  |
|  | >60 | 103 | *9.5* | | 167 | *17.1* | 270 | | *13.1* |  | 0.4 | *0.3-05* | *<0.001* |  |
| Gender | F | 384 | *35.4* | | 398 | *40.8* | 782 | | *38.0* | *0.012^(2)^* | 1 |  |  |  |
|  | M | 700 | *64.6* | | 578 | *59.2* | 1278 | | *62.0* |  | 1.261 | *1.05-1.5* | *0.012* |  |
| Type of kidney transplant | Single | 1003 | *92.5* | | 881 | *90.3* | 1884 | | *91.5* | *0.2^(2)^* | 1 |  |  |  |
|  | Double | 43 | *4.0* | | 47 | *4.8* | 90 | | *4.4* |  | 0.9 | *0.7-1.2* | *0.09* |  |
|  | Combined transplant | 38 | *3.5* | | 48 | *4.9* | 86 | | *4.2* |  | 0.8 | *0.6-0.9* | *0.06* |  |
| BMI^(3)^ | Normal weight | 684 | *63.1* | 527 | | *54.0* | 1211 | | *58.8* | *<0.001* | 1 |  |  |  |
|  | Overweight | 262 | *24.2* | 309 | | *31.7* | 571 | | *27.7* |  | 0.65 | *05-0.8* | *<0.001* |  |
|  | Obese | 40 | *3.7* | 74 | | *7.6* | 114 | | *5.5* |  | 0.42 | *0.3-06* | *<0.001* |  |
|  | Underweight | 72 | *6.6* | 50 | | *5.1* | 122 | | *5.9* |  | 1.1 | *0.8-1.6* | *0.6* |  |
|  | MD | 26 | *2.4* | 16 | | *1.6* | 42 | | *2.0* |  |  |  |  |  |
| Maximum PRA | 0-20 | 976 | *90.0* | | 860 | *88.1* | 1836 | | *89.1* | *0.4^(2)^* | 1 |  |  |  |
|  | 21-79 | 45 | *4.2* | | 56 | *5.7* | 101 | | *4.9* |  | 0.71 | *0.1-1.05* | *0.09* |  |
|  | >=80 | 20 | *1.8* | | 19 | *1.9* | 29 | | *1.4* |  | 0.8 | *0.4-1.6* | *0.6* |  |
|  | MD | 40 | *3.7* | | 44 | *4.5* | 84 | | *4.1* |  |  |  |  |  |
| Diagnosis | Glomerular nephropathies | 436 | *40.2* | | 389 | *39.9* | 825 | | *40.0* | *<0.001^(2)^* | 1 |  |  |  |
|  | Diabetic nephropathy | 24 | *2.2* | | 55 | *5.6* | 79 | | *3.8* |  | 0.39 | *0.24-0.64* | *<0.001* |  |
|  | Cystic nephropathies Congenital & Uropathies | 139 | *12.8* | | 150 | *15.4* | 289 | | *14.0* |  | 0.83 | *0.63-1.1* | *0.16* |  |
|  | Hypertensive Nephrosclerosis & Nephrovasculopathy | 47 | *4.3* | | 39 | *4.0* | 86 | | *4.2* |  | 0.95 | *0.58-1.6* | *0.8* |  |
|  | Other Kidney Diseases ^(4)^ | 438 | *40.4* | | 343 | *35.1* | 781 | | *37.9* |  | 1.2 | *0.95-1.3* | *0.16* |  |
| Dialysis vintage (years) | 1 | 86 | *7.9* | | 88 | *9.0* | 174 | | *8.4* | *0.2^(2)^* | 1 |  |  |  |
|  | 2 | 197 | *18.2* | | 160 | *16.4* | 357 | | *17.3* |  | 1.3 | *0.9-1.8* | *0.2* |  |
|  | 3 | 163 | *15.0* | | 134 | *13.7* | 297 | | *14.4* |  | 1.2 | *0.9-1.8* | *0.3* |  |
|  | 4 | 172 | *15.9* | | 140 | *14.3* | 312 | | *15.1* |  | 1.03 | *0.9-1.8* | *0.2* |  |
|  | >4 | 346 | *31.9* | | 350 | *35.9* | 696 | | *33.8* |  | 1.01 | *0.7-1.4* | *0.9* |  |
|  | MD | 120 | *11.1* | | 104 | *10.7* | 224 | | *10.9* |  | 1.8 | *0.8-1.8* | *0.4* |  |
| Delayed Graft Function | Yes | 276 | *25.5* | | 284 | *29.1* | 560 | | *27.2* | *0.4^(2)^* | 1 |  |  |  |
|  | No | 808 | *74.5* | | 692 | *70.9* | 1500 | | *72.8* |  | 1.2 | *0.99-1.5* | *0.06* |  |
| Case mix severity index at transplant | Standard | 213 | *19.6* | | 205 | *21.0* | 418 | | *20.3* | *0.04^(2)^* | 1 |  |  |  |
|  | Intermedium | 261 | *24.1* | | 224 | *23.0* | 485 | | *23.5* |  | 1.1 | *0.9-1.5* | *0.4* |  |
|  | Weak | 349 | *32.2* | | 269 | *27.6* | 618 | | *30.0* |  | 1.2 | *0.9-1.6* | *0.08* |  |
|  | Elevate | 261 | *24.1* | | 278 | *28.5* | 539 | | *26.2* |  | 0.9 | *0.7-1.2* | *0.4* |  |

MD: Missing Data; ^(1)^ t-test p-value; ^(2)^ Test di Kruskal-Wallis; ^(3)^ BMI according to the WHO classification; ^(4)^ Other kidney diseases include: tubulointerstitial nephropathies, renal cancer, acute renal failure and other kidney diseases.
